# An assistive listening device improves hearing following aneurysmal subarachnoid haemorrhage

**DOI:** 10.1111/ene.16240

**Published:** 2024-02-08

**Authors:** Ben Gaastra, Stuart Whyte, Bethan Hankin, Diederik Bulters, Ian Galea, Nicole Campbell

**Affiliations:** ^1^ Faculty of Medicine University of Southampton Southampton UK; ^2^ Department of Neurosurgery, Wessex Neurological Centre University Hospital Southampton Southampton UK; ^3^ Faculty of Engineering and Physical Sciences, Auditory Implant Service University of Southampton Southampton UK; ^4^ Faculty of Engineering and Physical Sciences University of Southampton Southampton UK

**Keywords:** assistive listening device, hearing, subarachnoid haemorrhage

## Abstract

**Background and purpose:**

Hearing impairment is common following aneurysmal subarachnoid haemorrhage (aSAH). Previous studies have demonstrated that auditory processing disorder (APD) is the primary underlying pathology. Assistive listening devices (ALDs) can be used to manage APD but have not been explored in aSAH. The aim of this study was to assess the benefit of an ALD for patients reporting hearing difficulty after aSAH.

**Methods:**

This was a prospective pilot single‐arm intervention study of an ALD for APD following aSAH. Patients who reported subjective hearing difficulty following aSAH were identified from the Wessex Neurological Centre aSAH database. Speech‐in‐noise was evaluated using the Bamford−Kowal−Bench (BKB) test under 60 and 65 dB noise conditions. BKB performance was compared with and without an ALD. Cognition was assessed using the Addenbrooke's Cognitive Examination‐III.

**Results:**

Fourteen aSAH patients with self‐reported hearing loss were included in the analysis. Under both noise conditions the ALD significantly improved BKB performance (60 dB, *Z* = −3.30, *p* < 0.001; 65 dB, *Z* = −3.33, *p* < 0.001). There was no relationship between cognition and response to the ALD.

**Conclusions:**

This study demonstrates the marked benefit of ALDs to manage APD following aSAH, regardless of cognitive status. This finding has implications for the management of this common yet disabling deficit which impacts quality of life and employment. A further trial of ALDs in this patient group is needed to test whether these large, short‐term benefits can be practically translated to the community for long‐term benefit when used at home.

## INTRODUCTION

Aneurysmal subarachnoid haemorrhage (aSAH) is a rare but devastating form of stroke caused by rupture of a cerebral artery aneurysm in the subarachnoid space. It is associated with significant morbidity and mortality with up to 40% of patients dying within 30 days [1]. Of the patients admitted to hospital around 60% return to independence and 10% survive in a dependent state at 1 year following haemorrhage [2]. Even survivors who achieve independence frequently suffer a range of persistent deficits including cognitive, auditory and psychological disabilities which impair quality of life and ability to return to work [3, 4, 5, 6, 7]. Compared to physical disability these deficits are not immediately obvious; however, the burden of these non‐visible disabilities following aSAH should not be underestimated.

Auditory complaints are common following aSAH with 20%–23% of patients reporting new onset hearing difficulty following haemorrhage [8, 9, 10]. Despite this they are clinically under‐recognised and are rarely formally diagnosed and treated. This is likely to be because patients do not classically report specific symptoms of hearing loss but rather report difficulty functioning in noisy environments which they frequently avoid. In a case–control study [10] it was shown that although aSAH patients had a normal pure tone audiogram (PTA) their speech‐in‐noise test scores were significantly worse versus controls, suggesting auditory processing difficulties—this is called auditory processing disorder (APD). Unlike peripheral hearing impairment, APD is caused by dysfunction or pathology within the central auditory nervous system and/or one of its modulatory pathways such as cognition [11]. APD classically presents as difficulty hearing in the presence of background noise despite a normal PTA.

In a second study, involving a larger cohort (*n* = 270) the finding of worse speech‐in‐noise perception following aSAH compared to controls was corroborated [3]. As cognition may play an important role in APD and it is common following aSAH [12] its contribution to hearing impairment was explored in the same study. It was shown that cognitive deficits, assessed by psychomotor reaction time, mediated a small but significant proportion (9.8%) of the effect of aSAH on hearing impairment [3]. Although cognitive deficits may contribute to hearing difficulty following aSAH, other factors such as direct central auditory nervous system damage play a key role. This is supported by the observation that there is increased iron deposition in the auditory cortex of aSAH patients with hearing difficulty compared to those without [10].

Understanding the underlying cause of hearing impairment following aSAH is essential to the management of this disability. Routine audiometry (which employs pure tones in the frequency range 0.25–8 Hz) fails to capture real‐world listening and speech perception, particularly in background noise and/or the presence of competing speech [13]. Adults presenting to audiology clinics with hearing difficulty are typically dismissed if they have normal PTA results, potentially leading to frustration as they continue to struggle in less favourable acoustic environments with poor signal‐to‐noise ratio (SNR).

There are a number of assistive listening devices (ALDs), currently not available in the UK National Health Service for this patient population, that can be considered for those with a normal PTA but speech‐in‐noise difficulty. The most commonly used is wireless remote microphone (WRM) technology consisting of a microphone transmitter and receiver(s), with the talker's voice being relayed directly to the receiver and so improving the SNR by 10–15 dB or more [13]. Small ear level receivers are worn on each ear by the person with APD, which function together with a WRM worn by the communication partner. Sound is picked up by the microphone and streamed directly (and wirelessly) to the receivers, that is, directly to the ears. This helps to cut out the background noise making hearing in the presence of competing sound easier. Traditionally these devices have been used in classroom/lecture hall or ‘listening to one person in a car’ type settings where one person is talking and the other listening. Although mostly used for children with APD, Koohi et al. [14] have reported that the technology can also be beneficial in adults, including those with a normal PTA but difficulty hearing after a stroke. In this study the focus is on the potential benefit of an ALD using WRM technology for patients with a normal PTA but reporting difficulty with hearing speech, particularly speech‐in‐noise. This technology has been selected because benefit has already been established for children with APD and there is also some evidence that it is beneficial for adults after stroke types other than aSAH [14].

The aim of this study was (i) to assess the benefit of an ALD using WRM technology for patients with a normal PTA but reporting hearing difficulty after aSAH; and (ii) to evaluate whether cognitive deficits following aSAH influence the response to an ALD.

## METHODS

This prospective pilot single‐arm intervention study of an ALD using WRM technology for APD following aSAH received both national (REC 12/SC/0666) and local (ERGO 5650) ethical approval. All participants gave informed consent. The study is reported according to the CONSORT statement extension to pilot and feasibility trials [15].

Patients were identified from the Wessex Neurological Centre Subarachnoid Haemorrhage database. Patients aged 18 years or older with a diagnosis of aSAH were eligible for inclusion if they had reported new onset hearing difficulty following aSAH on routine assessment with the Subarachnoid Haemorrhage‐specific Outcome Tool (SAHOT) [9].

All eligible patients were contacted by letter inviting them to participate in the study and respondents were invited for formal assessment at the University of Southampton.

### Assessment

Patients initially underwent otoscopy, tympanometry and pure tone audiometry. Individuals were excluded if they had an abnormal otological examination. The pure tone thresholds for 250, 500, 1000, 2000 and 4000 Hz were averaged and those with an average of >25 dB hearing level were excluded. A threshold of >25 dB hearing level in either ear was chosen to account for age‐related hearing loss in this patient demographic [16, 17].

### Speech‐in‐noise testing rig

The speech‐in‐noise testing rig used in this study is an adaptation of that used by the Europäische Union der Hörakustiker (EUHA) and Husstedt et al. in which the laboratory was calibrated to record the sound pressure as dB sound pressure level (SPL) [18, 19]. The EUHA [18] speech‐in‐noise testing rig was adapted to use dBA measures which emulate a situation where people are listening to speech from a distance. The background for this is that the frequency range between 1 and 4 kHz is important for speech intelligibility, with perception up to 8 kHz also important for fricatives [20]. As humans have different levels of sensitivity to different frequencies of sound, the dBA weighting reduces the SPLs at frequencies below 1000 Hz and frequencies above 8000 Hz [21]. In the frequency range important for understanding speech, dB SPL and dBA are nearly equivalent and within the acceptance limits of a calibrated class 2 sound level meter [21].

**FIGURE 1:**
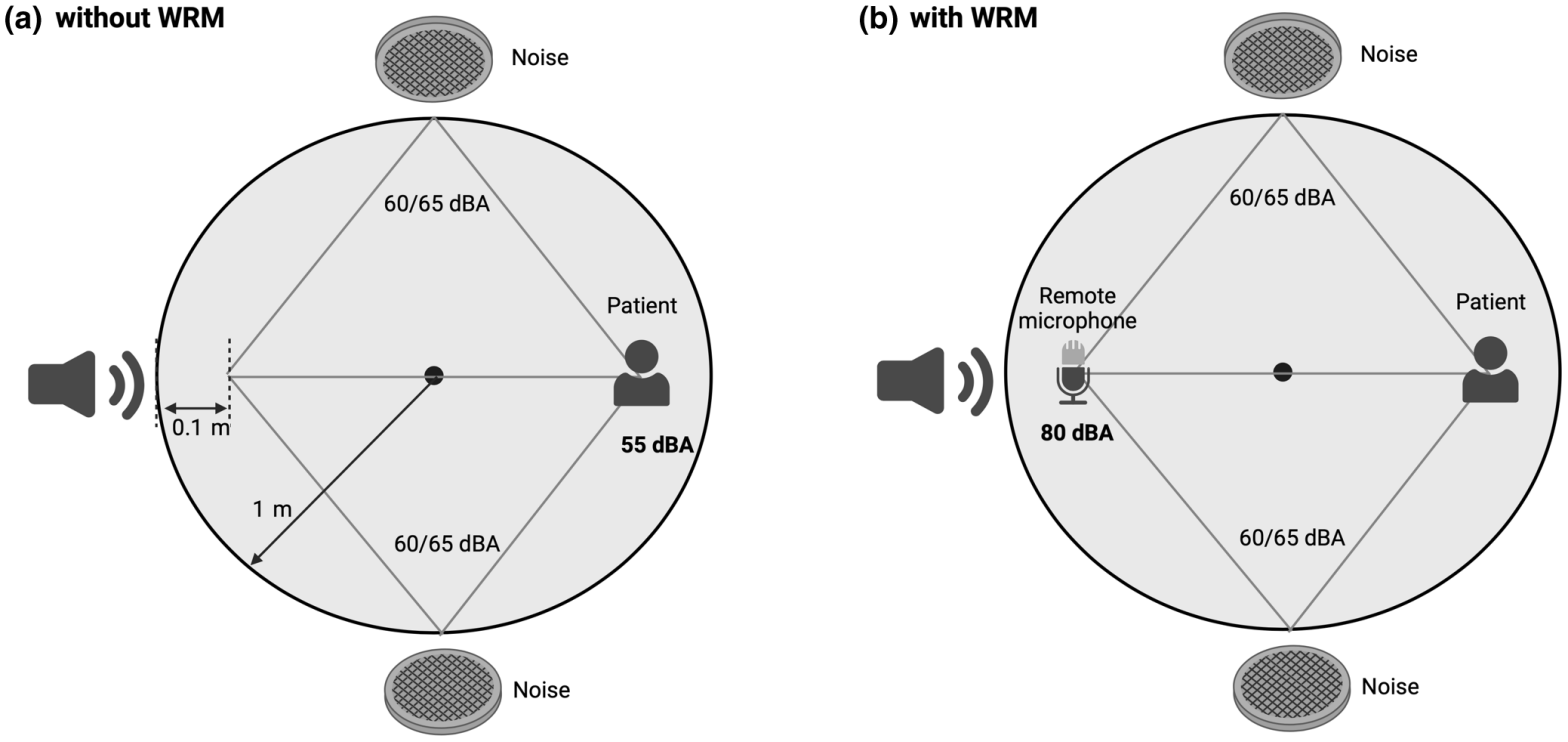
Diagram of testing rig with and without WRM demonstrating speech signal. Created with Biorender.com.

The rig setup consisted of three loudspeakers within a circle of 1 m radius (Figure 1). Speech signals were delivered by the Soundbyte Solutions Parrot Touch system. The Parrot speech discrimination tester is widely used in audiological centres. Two Soundbyte Solutions Noise Cubes presented multi‐talker babble in the sound field at 60 dBA or 65 dBA. Noise was presented from approximately ±45° from the perspective of the patient, as well as from the perspective of the speaker. This rig has the advantage of being a mobile set‐up, which has practical implications for clinical trials.

To test speech‐in‐noise without the WRM a speech signal of 60 dBA was presented and, if necessary, adjusted in 1 dB steps so that the signal reaching the patient was 55 dBA (Figure 1). This value represented listening to conversational speech and at a distance of approximately 3 m. To test speech‐in‐noise with the WRM both the patient and the device were positioned in front of the loudspeaker presenting the speech signal (Figure 1). At the remote microphone the speech signal was set at 80 dBA The loudspeakers presenting the noise signal were placed such that they were equidistant to the listener and to the WRM.

The Bamford−Kowal−Bench (BKB) speech test [22] was used to evaluate the patient's ability to discriminate speech‐in‐noise at fixed −5 dB (60 dB noise condition) and −10 dB (65 dB noise condition) SNRs. Each BKB sentence was scored based on the percentage of correctly repeated key words. At both SNRs the BKB test was repeated with and without the ALD using WRM technology which consisted of a Phonak Roger On transmitter and a Phonak Roger NeckLoop receiver.

Cognition and hearing‐related quality of life were assessed using the Addenbrooke's Cognitive Examination‐III (ACE‐III) [23] and the Hearing Handicap Inventory for Adults (HHIA) [24], respectively. An ACE‐III score ≤88 was used to define cognitive impairment [25] with a score of ≤76 indicating moderate dementia [26]. An HHIA score ≥44 was used to define severely impaired hearing‐related quality of life. Baseline demographic and clinical data were collected from participant health records including age, sex, time following aSAH, World Federation of Neurological Surgeons (WFNS) score [27] and aneurysm treatment modality (endovascular/surgery).

Patients were also asked to answer, by scoring from 0 (no effect) to 100 (large effect), two questions, (i) ‘Was the listening device beneficial in the speech‐in‐noise test?’ and (ii) ‘Did the listening device make listening less of an effort?’, and to provide free verbal or textual feedback.

### Analysis

Data were tested for normality using the Shapiro–Wilk test. BKB test performance with and without the ALD was compared using the non‐parametric paired Wilcoxon signed rank test. To assess whether cognition affected response to the ALD the relationship between ACE‐III and change in BKB performance with and without the ALD, controlling for age, was assessed using partial correlation analysis. Partial correlation analysis, controlling for age, was also used to assess the relationship between ACE‐III and HHIA scores. Pearson product–moment partial correlation analysis was used for parametric data and Spearman rank partial correlation analysis for non‐parametric data. As a sensitivity analysis the partial correlation analysis was repeated additionally controlling for time after aSAH.

All analyses were performed using SPSS Statistics (version 29.0, IBM Corporation). Alpha was 0.05.

## RESULTS

**FIGURE 2:**
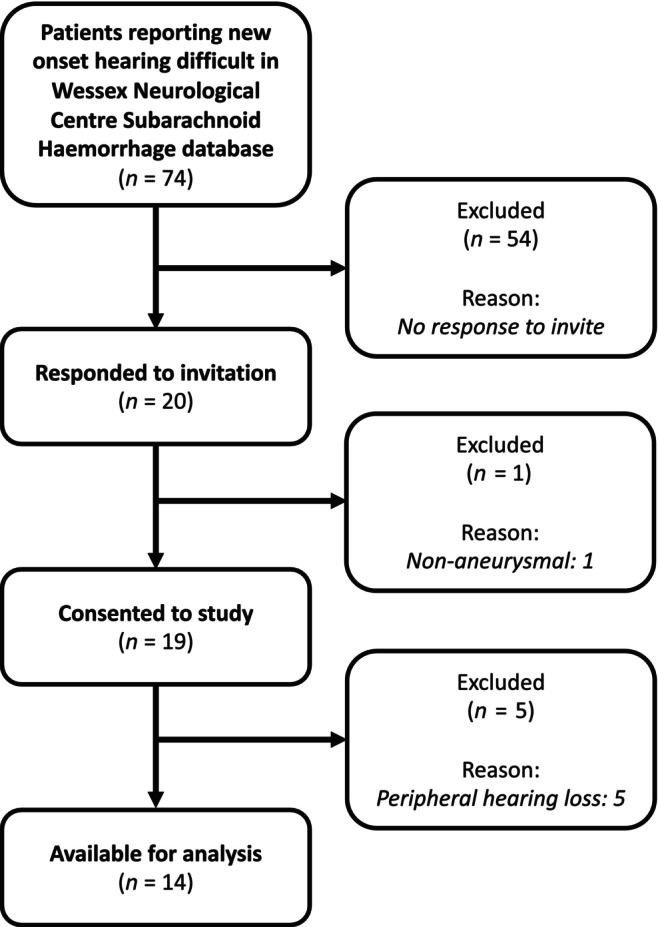
Flowchart of patients included in the study.

Seventy‐four patients with new onset hearing difficulty were identified from the Wessex Neurological Centre Subarachnoid Haemorrhage database and invited to participate. Twenty patients responded and attended for assessment. One patient was excluded as the subarachnoid haemorrhage was subsequently identified to be non‐aneurysmal in nature. Following PTA a further five patients were excluded as they had evidence of peripheral hearing loss and were referred for follow‐up by their general practitioner (see Figure 2 for flowchart of patient inclusion).

Table 1 summarizes the demographics of the included patients (*n* = 14). The mean patient age was 57.4 years with mean time after aSAH of 38.3 months. In keeping with expected demographics for aSAH the majority were female (79%), treated endovascularly (64%) and had a WFNS grade of 1 (50%), which means that patients were fully awake, oriented and responsive to commands on admission to hospital after resuscitation. The Shapiro–Wilk test showed that time after aSAH, HHIA and ACE‐III scores did not differ significantly from a normal distribution.

**TABLE 1:** Demographics of patients included in the analysis.

Age (years)	
Mean ± SD	57.4 ± 11
Sex	
Male	3 (21%)
Female	11 (79%)
Treatment	
Surgical	5 (36%)
Endovascular	9 (64%)
WFNS score	
1	7 (50%)
2	2 (14%)
3	2 (14%)
4	3 (21%)
5	0 (0%)
Time after aSAH (months)	
Mean ± SD	38.3 ± 24.1

Abbreviation: aSAH, aneurysmal subarachnoid haemorrhage; SD, standard deviation; WFNS, World Federation of Neurosurgical Surgeons.

**FIGURE 3:**
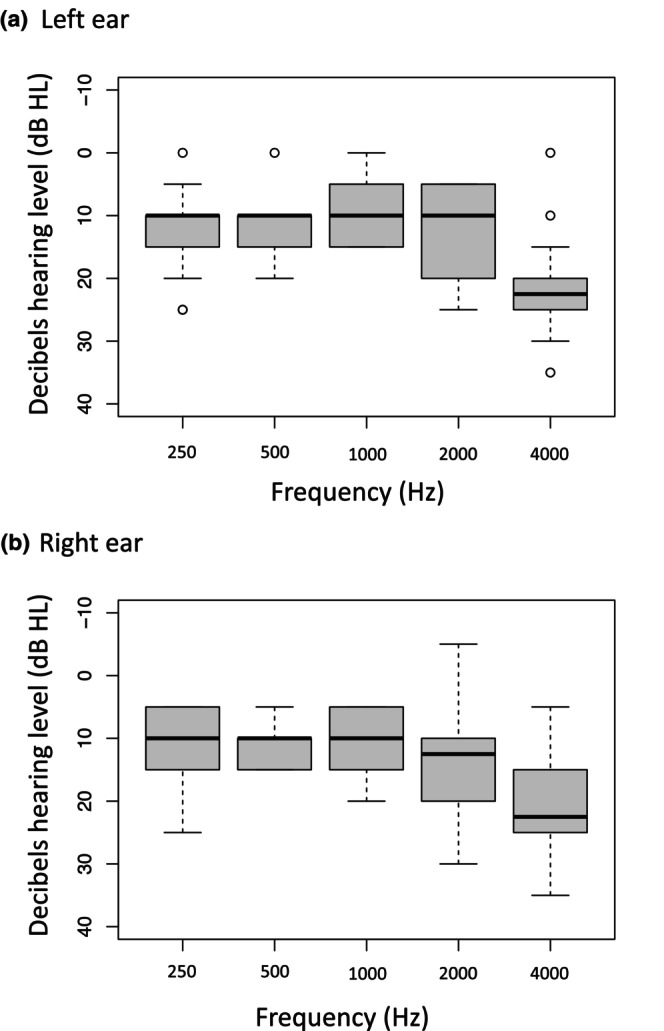
Mean PTA thresholds for the 14 patients meeting the inclusion criteria: (a) left ear and (b) right ear.

Table 2 details the mean PTA, cognitive (ACE‐III) and hearing‐related quality of life (HHIA) assessments for all included patients. Eight patients (57%) were classified as having severely impaired hearing‐related quality of life and nine patients (64%) were cognitively impaired. Using Pearson product−moment partial correlation analysis, controlling for age, the HHIA and ACE‐III scores were significantly negatively correlated (*r* = −0.610, *p* = 0.027). Figure 3 summarizes the mean PTA thresholds for each ear.

**TABLE 2:** Heat map of PTA, cognitive (ACE‐III) and hearing‐related quality of life (HHIA) assessment for all patients.

Participant	Mean PTA left ear	Mean PTA right ear	ACE‐III score	HHIA score
1	14	9	76	82
2	16	16	76	98
3	12	16	90	58
4	12	10	89	68
5	11	10	98	10
6	15	9	87	28
7	15	23	82	60
8	14	16	84	12
9	7	4	85	68
10	7	14	96	14
11	21	17	87	4
12	8	9	86	78
13	19	17	98	54
14	18	22	87	38

*Note*: Grey shading signifies severely impaired hearing‐related quality of life (defined as ≥44) and cognitive impairment (defined as ACE‐III ≤88).

Abbreviations: ACE‐III, Addenbrooke's Cognitive Examination III; HHIA, Hearing Handicap Inventory for Adults; PTA, pure tone audiogram.

### Comparison of performance with and without ALD

**FIGURE 4:**
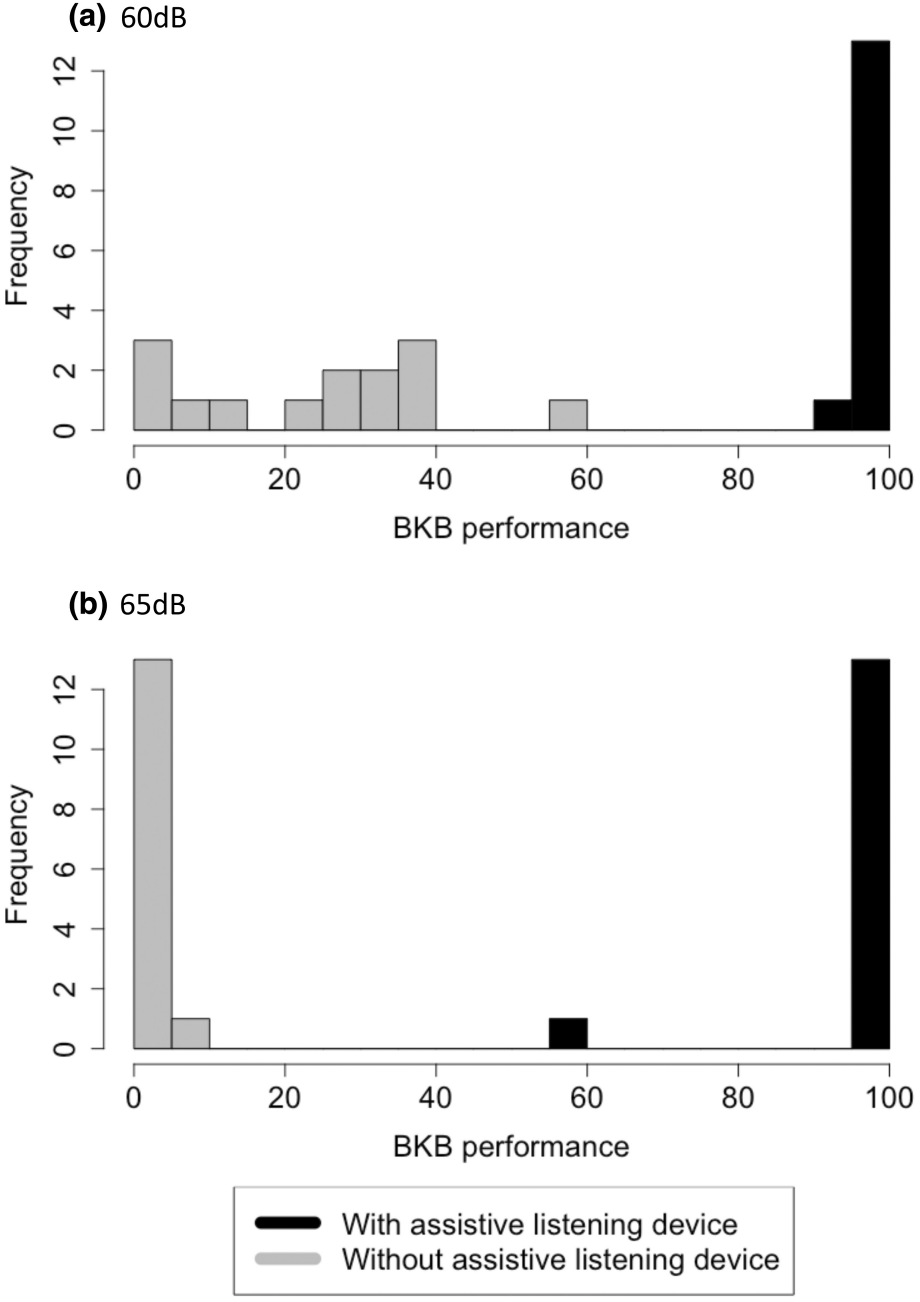
Histograms of BKB score with and without assistive listening device in (a) 60 dB and (b) 65 dB noise conditions.

The BKB scores at 60 dB (with ALD) and 65 dB (with and without ALD) significantly differed from a normal distribution. There was an improvement in BKB scores in all participants in both noise conditions. The improvement with the ALD was marked with a complete separation of the data distributions (with and without the ALD) observed in the frequency histograms (Figure 4a,b).

In the 60 dB noise condition, the mean BKB words correctly repeated without the ALD was 25% (standard deviation [SD] ± 0.17), which significantly increased to a mean of 99% (SD ± 0.02) with the ALD (*Z* = −3.30, *p* < 0.001, Figure 4a). In the 65 dB noise condition the mean BKB words correctly repeated without the ALD was 1% (SD ± 0.02), which again increased significantly to a mean of 97% (SD ± 0.11) with the ALD (*Z* = −3.33, *p* < 0.001, Figure 4b).

### Relationship between response to an ALD and cognition

The response to an ALD did not differ significantly from a normal distribution under the 60 dB noise condition but did under the 65 dB noise condition. There was no relationship between cognition, as assessed by the raw ACE‐III score, and response to the ALD under either noise condition using the Spearman rank partial correlation analysis (BKB 60 dB, *r* = −0.478, *p* = 0.098; BKB 65 dB, *r* = −0.150, *p* = 0.626), controlling for age. In the sensitivity analysis there was no change in the significance of the results if the analysis was repeated controlling for time after aSAH.

### Patient feedback

The mean response to the question ‘Was the listening device beneficial in the speech‐in‐noise test?’ was 91 out of 100 (SD ± 14). The mean response to the question ‘Did the listening device make listening less of an effort?’ was 85 out of 100 (SD ± 24). All participants left very positive feedback. Comments included ‘could not believe the change’, ‘astounded’ and ‘amazed at the difference’. Several reported the ALD to be ‘potentially life changing’ and ‘will make life easier’. One participant reported ‘I feel I could hear’, another said it ‘made hearing a lot clearer’ and yet another ‘incredible’. They also reported the ALD could potentially alleviate social anxiety and fatigue and facilitate life at the workplace and outside.

## DISCUSSION

In this pilot study it was demonstrated that an ALD using WRM technology significantly improved auditory outcome after aSAH in patients with difficulty hearing, particularly speech‐in‐noise, despite having a normal PTA. The effect size was very large such that in noisy conditions almost all patients went from discriminating no words without the device to recognising all words with it. This effect was seen at a mean time from aSAH of over 3 years suggesting the benefit persists in the long term and is not limited to the acute phase after haemorrhage. The degree of this therapeutic effect was not influenced by cognition suggesting a benefit of ALDs even in the presence of cognitive impairment. Patient feedback highlighted that the WRM was not only beneficial in the speech‐in‐noise test but also reduced the effort of listening. Individual feedback was overwhelmingly positive highlighting the potential benefit of WRMs in both the home and work environment. Together these results support larger trials of ALDs to manage hearing impairment following aSAH.

Although new onset hearing difficulty is common following aSAH, affecting up to 23% of survivors [10], impairing quality of life [28] and leading to unemployment [29], it is not routinely assessed and there are currently no recommendations for its management. It has previously been demonstrated that APD underlies hearing difficulty following aSAH [3, 10] and this understanding of the pathology is essential to guide ongoing management. There is no gold standard for the management of APD but a number of strategies can be considered including modification of the listening environment, technology (such as WRM, low‐gain hearing aids or a combination), auditory training and compensatory strategies [11]. WRM technology is a promising management strategy for APD and has already been shown to be beneficial following ischaemic and haemorrhagic stroke [14, 30]. In this study the first evidence is provided that an ALD using WRM technology significantly improves hearing impairment following aSAH. This therapeutic strategy has the potential to benefit a significant proportion of aSAH survivors by improving quality of life and relieving the socioeconomic burden of aSAH through return to work.

The focus of this study was the use of an ALD using WRM technology in the standard way (so not one of ‘table’, ‘pointing’ or ‘streaming’ modes) during the single visit to an audiology clinic. Further research is needed to look at outcomes with the newer digital remote microphone technology features including ‘table mode’ where the microphone (changing from directional to omnidirectional mode) is placed on the table and picks up speech from a small group around a table, ‘pointing mode’ where the microphone is pointed in the direction of the primary speech signal, or ‘streaming mode’ set to wirelessly stream from a mobile phone, laptop or TV audio output. Further research is also needed to explore how patients experience use of the technology in the real world, that is, in their free‐living environment. It would also be of value to look at how the outcomes with the ALD used here compare to low‐gain hearing aids with directional microphones, narrow‐band beam‐forming directionality and multiple speaker access and also a combination of both WRM and low‐gain hearing aids [13]. More recently and in countries such as the United States over‐the‐counter hearing aids and personal sound amplification products have also become available and warrant further research.

Auditory processing disorder (APD) is caused by dysfunction or pathology within the central auditory nervous system or the network of modulatory pathways which interact with it such as cognition. Cognitive deficits are common following aSAH with a prevalence of up to 70% [12] and it has been demonstrated previously that they significantly contribute to APD following haemorrhage [3]. It was therefore assessed whether cognition, assessed by the raw ACE‐III score, influenced response to the ALD as this would have implications for its future therapeutic use. In this small cohort no relationship between cognition and benefit from the ALD was observed, suggesting that WRM technology would be an appropriate treatment for APD following aSAH regardless of cognitive status. In this cohort the majority of patients with cognitive impairment (7/9) were classed as having mild dementia (ACE‐III >76) and caution should be taken when extending these results to patients with more significant cognitive impairment.

The novel testing rig developed in this study has two major advantages: (i) it is able to effectively assess speech‐in‐noise in the non‐laboratory setting; (ii) it is mobile and easily set up. Hence it could be deployed to facilitate comprehensive and easy access to investigation of potential APD (once peripheral hearing impairment has been ruled out) in community clinical service or multi‐centre clinical trial settings. This is particularly relevant to the aSAH population who experience a wide spectrum of morbidity, including fatigue [5], which may limit their ability to attend hearing assessments in a laboratory environment in a tertiary centre.

A strength of this study was the consideration of speech‐in‐noise perception in addition to standard pure tone audiometry. A fixed BKB test was performed in a laboratory setting at a single point in time to demonstrate the benefit of the ALD. Future studies are required to confirm the benefit of this technology in a free‐living environment. Previous studies in children have demonstrated that its benefits increase after prolonged use [31] and consequently further studies are required to evaluate the full benefit of ALDs in the long‐term following aSAH. With the fixed BKB test used in this study there was evidence of both a floor and ceiling effect with a significant number of patients scoring 0% without the ALD, especially in the 65 dB noise condition, and improving to 100% with the ALD. An adaptive BKB test may avoid this and more accurately quantify the benefit of an ALD following aSAH. The use of additional test stimuli such as digits may reduce the cognitive/language load and broaden test use to patients with significant cognitive deficits.

There were a number of limitations of this study. First, the sample size was small, although for the chosen study outcomes more than adequate given the size of the effect observed. Larger studies are required to investigate the practicality of its use in the community and effects on quality of life. Secondly, although all patients reporting hearing difficulty were invited for inclusion in this study, some responder bias is likely to have occurred in favour of the most motivated and least physically disabled patients since the study required detailed in‐person assessment. Although future studies should strive to include a wide range of survivors of aSAH to confirm the benefit of ALDs across the recovery spectrum, this type of bias is impossible to completely eliminate in an intervention study of a promising new device. Finally, peripheral hearing deficits are known to coexist with APD following aSAH, not least due to age‐related peripheral hearing loss [32]. In this study 5/19 patients (26.3%) assessed had coexistent peripheral hearing loss and were excluded. Future work is required to assess the optimum management strategy for patients with both central and peripheral hearing impairment following aSAH.

In conclusion, this study demonstrates the marked benefit of ALDs to manage APD following aSAH, regardless of cognitive status. This finding has major implications for the management of this common yet disabling deficit which impacts quality of life and employment. A further trial of ALDs in this patient group is needed to test whether these large short‐term benefits can be practically translated to the community for long‐term benefit when used at home.

## AUTHOR CONTRIBUTIONS

**Ben Gaastra:** Conceptualization; writing – original draft; methodology; writing – review and editing; formal analysis; data curation. **Stuart Whyte:** Conceptualization; writing – original draft; writing – review and editing; methodology; supervision. **Bethan Hankin:** Writing – review and editing; formal analysis; data curation. **Diederik Bulters:** Conceptualization; writing – review and editing; supervision; methodology. **Ian Galea:** Conceptualization; writing – review and editing; supervision; methodology. **Nicole Campbell:** Conceptualization; writing – original draft; writing – review and editing; supervision; data curation; methodology.

## FUNDING INFORMATION

BG is funded by the National Institute for Health Research (NIHR), Guarantors of Brain and Institute for Life Sciences at the University of Southampton.

## CONFLICT OF INTEREST STATEMENT

The authors have stated explicitly that there are no conflicts of interest in connection with this article.

## Data Availability

The data that support the findings of this study are available from the corresponding author upon reasonable request.
